# Supplementary material for: Differentiation of human adult-derived stem cells towards a neural lineage involves a dedifferentiation event prior to differentiation to neural phenotypes
Source: Sci Rep. 2021 Jun 8;11:12034. doi: 10.1038/s41598-021-91566-9 (PMC8187441; doi:10.1038/s41598-021-91566-9)
Supplement: Supplementary file 1 — Supplementary Figures. [file 41598_2021_91566_MOESM1_ESM.docx]

**Supplementary information**

Differentiation of human adult-derived stem cells towards a neural lineage involves a dedifferentiation event prior to differentiation to neural phenotypes

**Authors**: Carlos Bueno^1*^, Marta Martínez-Morga^2^, David García-Bernal^3^, José M Moraleda^3^ and Salvador Martínez^1^.

^1^ Instituto de Neurociencias de Alicante (UMH-CSIC), San Juan, Alicante, 03550, Spain.

^2^ Department of Human Anatomy and Institute of Biomedical Research (IMIB), University of Murcia, Faculty of Medicine, Murcia, 30800, Spain.

^3^ Internal Medicine Department and Hematopoietic Transplant and Cellular Therapy Unit, Institute of Biomedical Research (IMIB), University of Murcia, Faculty of Medicine, Murcia, 30800, Spain.


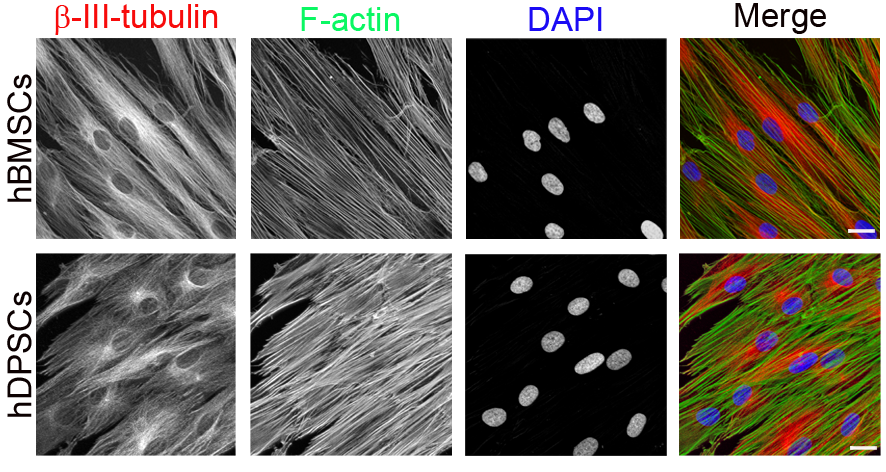


**Figure S1. Morphology of hBMSCs and hDPSCs cultured in basal media**. During interphase, undifferentiated hBMSCs and hDPSCs presented a fibroblast-like morphology with actin microfilaments and β-III tubulin microtubules oriented parallel to the cell’s longitudinal axis and an ellipsoidal nucleus, often located in the centre of the cell. Scale bar: 25 μm.

**
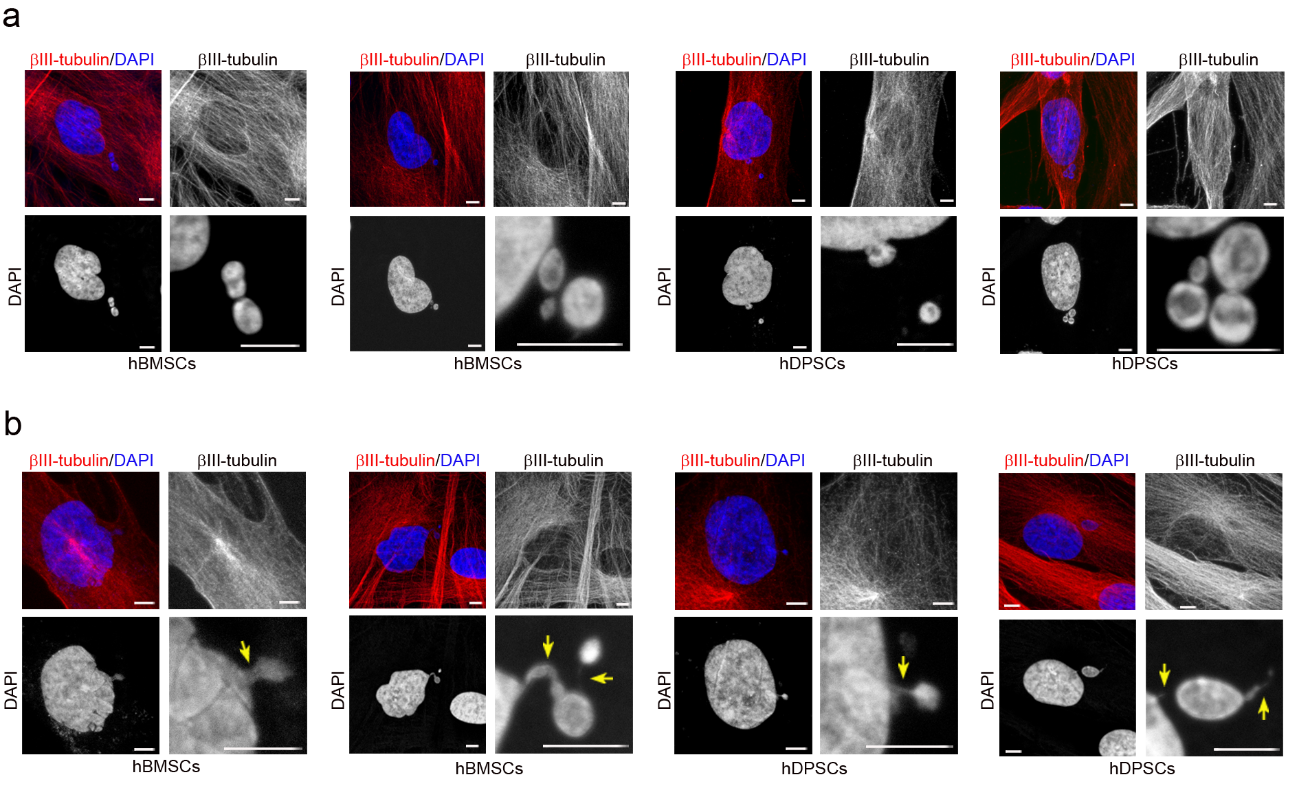
**

**Figure S2. Cytoplasmic DNA-containing structures**. Cytoplasmic DNA-containing structures with a spherical or ovoid shape (**a**) and some appeared to be connected to the main body of the nucleus by thin strands of nuclear material (**b**, yellow arrows). Scale bar: 5 μm.
